# Exploring the Impact of Health Literacy on Fertility Awareness and Reproductive Health in University Students—A Systematic Review

**DOI:** 10.3390/healthcare13182342

**Published:** 2025-09-17

**Authors:** Viktória Prémusz, Melese Dereje Mesfin, Leman Atmaca, Shalini Chauhan, Zoltán Tándor, Lili Andrea Bodor, Ákos Várnagy, Dahabo Adi Galgalo

**Affiliations:** 1 Doctoral School of Health Science, Faculty of Health Science, University of Pécs, H-7621 Pécs, Hungary; mesfin.melese@edu.pte.hu (M.D.M.); atmaca.leman@edu.pte.hu (L.A.); shalini.chauhan@pte.hu (S.C.); tz@med.unideb.hu (Z.T.); bodor.andrea@pte.hu (L.A.B.); bwqi8p@pte.hu (D.A.G.); 2 National Laboratory on Human Reproduction, University of Pécs, H-7622 Pécs, Hungary; varnagy.akos@pte.hu; 3 Faculty of Health Sciences, University of Pécs, H-7621 Pécs, Hungary; 4 Centre for Assisted Reproduction, Clinical Centre, University of Debrecen, H-4032 Debrecen, Hungary; 5 Department of Obstetrics and Gynaecology, Medical School, University of Pécs, H-7624 Pécs, Hungary; 6 Directorate for Human Reproduction, National Directorate General for Hospitals, H-1125 Budapest, Hungary

**Keywords:** health literacy, fertility awareness, reproductive health, students, sexual education

## Abstract

**Background/Objectives:** Health literacy has an impact on students’ reproductive health. Therefore, the objective of our study is to systematically examine, identify, and summarize all research on the role of health literacy in fertility awareness and reproductive health among university students in order to understand how health literacy influences reproductive health outcomes in this population. **Methods:** Using the PRISMA guidelines, a comprehensive systematic search was conducted using electronic databases, such as PubMed, EMBASE, CINAHL, Scopus, and Google Scholar. The protocol was registered in the Prospective Register for Systematic Reviews (PROSPERO, CRD 42024566268). All studies were imported into EndNote software and screened using a two-level title/abstract screening process. The included studies were narratively summarized. **Results:** The database search identified 1360 articles; 116 duplicates were removed, and thus, 1244 were initially screened, leading to 1133 exclusions. A total of 111 articles underwent full screening, and 94 were then excluded. A total of 14 articles were included for data extraction. Health literacy impacts university students’ behaviour, fertility awareness, and reproductive health knowledge. Students with higher health literacy show more understanding of critical topics such as fertility and emergency contraception. Socioeconomic factors play a crucial role in shaping health literacy and reproductive choices, while gender disparities highlight the need for targeted educational interventions, particularly for male students. Effective educational programs have been shown to enhance health literacy. Additionally, technology integration serves as a valuable tool for disseminating reproductive health information. Cultural context also plays a vital role in influencing health literacy. **Conclusions:** The findings of this study emphasize the importance of comprehensive strategies to enhance health literacy among university students, and future research should focus on developing and evaluating targeted educational programs that address gender disparities and socioeconomic factors influencing health literacy.

## 1. Introduction

Reproductive health is a state of complete physical, mental, and social well-being of the reproductive system and all its functions, and it is a critical feature of human growth [1]. This involves menstrual problems, cervical screening, fertility, contraceptive use, chronic illness, and sexually transmitted infections [1,2,3,4]. Reproductive health is essential to general health and well-being, especially during young adulthood, when significant physical, social, and psychological changes occur. Between the ages of 18 and 25, in particular, high school students who share important developmental, social, and contextual similarities with university students can go through difficult times as they begin to make decisions about their relationships, habits, and health [5]. During this period, they are at risk of encountering many reproductive health-related problems, such as unwanted pregnancies, sexually transmitted diseases, inappropriate or irregular use of contraceptive methods, and lack of awareness or access to reproductive and sexual health services [6]. To address these problems and utilize health services, individuals must be able to access, understand, and use health-related information [7].

Fertility awareness is an essential aspect of reproductive health, especially among university students who are going through the period of life marked by significant physical, emotional, and social changes [8,9]. Fertility awareness includes, among other things, knowledge about the menstrual cycle, ovulation, and the biological factors that influence reproduction [10,11,12]. Awareness of these topics is critical because it leads to informed decisions regarding family planning, contraception, and overall reproductive health.

Health literacy is the ability of individuals to access, understand, and utilize health information and services to make decisions about their health. It is a major social determinant of health and is a key factor in an individual’s health-related behaviour and the outcomes achieved [13,14]. People with low levels of reproductive health knowledge face challenges in managing their health effectively, and this can lead to poor health outcomes [13,15]. Low health literacy can lead to misunderstandings about fertility, resulting in unintended pregnancies or inadequate preparation for parenthood [16]. Health literacy is effective in helping an individual understand their sexual rights and consent, obtain information about sexually transmitted diseases, utilize appropriate protection methods and patterns, and access reproductive health services when necessary [17,18,19]. Improving health literacy is critical for empowering individuals, promoting equity, and improving health outcomes [15].

A study by D’Anci and Holschuh found a significant relationship between health literacy levels and reproductive health outcomes. It revealed that college students with higher health literacy levels show better reproductive health knowledge and engage in safer sexual practices [20]. Students who possess greater reproductive health literacy are more proactive in seeking information and services related to sexual health and highlighted the urgent need for effective health education programs in universities that not only enhance health literacy but also empower students to make informed decisions regarding their reproductive health [21,22,23,24,25]. Some barriers to health literacy continue to pose challenges for young adults [26]. Misinformation and limited access to reliable sources significantly hinder health literacy among students in different demographic areas. Addressing the barriers to reproductive health literacy and ensuring that students navigate available health literacy and reproductive health behaviours is critical [27,28].

Basic knowledge or having a solid understanding of reproductive health is crucial for sexually active university students. This knowledge empowers them to make informed decisions about their reproductive choices, ultimately protecting and enhancing their sexual health [21,29]. These students are often at the threshold and face unique challenges that can impact their health literacy and, consequently, their fertility awareness. Some factors, like lifestyle choices, academic stress, and access to health education resources, can influence their understanding of reproductive health issues. A healthy lifestyle which includes a balance diet and regular physical exercises, can enhance cognitive function and overall well-being, which improves an individual’s ability to process and understand information. Higher levels of stress can impair cognitive functions, making it difficult for students to focus on and retain information about reproductive health, while the presence of comprehensive and easily accessible health education and understanding resources can significantly improve student knowledge and their understanding of reproductive health issues. Reproductive health literacy is essential, especially for students who are easily shaped by the pressures of society at university age and who have not previously received sufficient sexual health education. It helps individuals access existing health services and make informed decisions.

While several studies have examined young adults’ attitudes, understanding, and access to reproductive health treatments, it is less clear what the role of students’ health literacy in fertility awareness and reproductive health is. Understanding this association within the student population is lacking because existing research focuses primarily on adolescents or general health outcomes. The purpose of this systematic review is to explore the intersection of health literacy, fertility awareness, and reproductive health in this demographic, which is vital for developing targeted educational interventions and support systems. To improve reproductive health by focusing on health literacy in the future, this systematic review intends to guide future research, policy developments, and educational strategies by identifying patterns, gaps, and implications.

### Conceptual Framework

**Figure 1:**
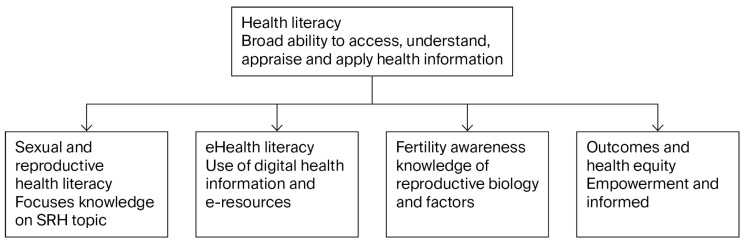
Conceptual framework.

This systematic review uses a conceptual framework that distinguishes and integrates different forms of literacy relevant to sexual and reproductive health, acknowledging their unique contributions to health. Health literacy is defined as an individual ability to access understand, appraise, and apply health information to make informed decisions promoting health and well-being as per the WHO guidelines, 2024. Sexual and reproductive health literacy refers specifically to the knowledge and competencies related to sexual and reproductive health topics, including contraception, fertility, and disease prevention [30]. This focused literacy domain is necessary to support informed reproductive choices, reduce unintended pregnancies, and promote sexual health [31]. E-Health Literacy is defined as the ability to seek, find, understand, and appraised health information from electronic sources [32]. With growing reliance on digital health resources, e-Health literacy enables individuals to effectively utilize technology for health management as per the WHO guidelines, 2024. Fertility awareness is the knowledge and understanding of human reproductive biology and factors affecting fertility [33]. It supports individuals in making informed reproductive decisions and aligns with broader reproductive health (see Figure 1).

## 2. Materials and Methods

### 2.1. Sources

This systematic review protocol was registered in the International Prospective Register for Systematic Reviews (PROSPERO) on 18 July 2024 (registration number: CRD 42024566268). The review adhered to standards set by the Reporting Items for Systematic Reviews and Meta-Analyses (PRISMA) guidelines [34].

### 2.2. Eligibility Criteria

A complete literature search of all studies published in English in peer-reviewed journals, focusing on the role of health literacy among university students in fertility awareness and reproductive health, was carried out. Studies were included if they met the following criteria: (1) if the study focused on the level of fertility awareness and reproductive health (FA) among university, college, and high school students; (2) if the authors employed an FA-specific measure or provided a detailed description of the questions assessed; and (3) if the paper used quantitative data related to FA (e.g., awareness of giving birth, fertile age, definition of infertility, factors affecting fertility, lifestyle risk factors, age-related fertility decline, and success rates of medically assisted reproduction treatments). Exclusion criteria included discussion papers, theses, dissertations, editorials, commentaries, and studies that lacked specific measures or detailed descriptions of FA questions. Papers like dissertations were excluded because they could compromise the reliability of the findings due to limited peer review. Some studies, such as case studies and case series, were also excluded because of insufficient data and unclear methodology. Lack of transparency in methodology, study design, and sample size was considered low-quality evidence.

### 2.3. Search Strategy

A comprehensive search was conducted by research team members (VP, DAG, LE, MD, and SC) independently using predefined keywords and subject headings in major electronic databases, including PubMed, Scopus, CINAHL, Google scholar, and EMBASE. Electronic searches were conducted in addition to looking through the included publications’ reference lists and contacting subject-matter experts for information. There was no restriction on the publication date; the search was carried out between 29 March and 1 April 2025 in order for the results to be comprehensive. The search terms used for searching were as follows: (College Student* OR Student* OR University OR Universities OR Higher Education OR Campus AND Health Literacy AND Fertility OR Fertility Awareness OR Fertility Knowledge OR Fertility Intention* OR Fecundability OR Fecundity OR Fertility Incentives OR Fertility Incentive OR World Fertility Survey OR Fertility Determinants OR Fertility Preferences OR Fertility Preference OR Reproductive Behaviour OR Childbearing OR Childbirth OR Reproductive Health Knowledge OR Fertility Education OR Conception Knowledge OR Reproductive Life Span OR Family Planning OR Fertility Literacy OR Reproductive Health). The search strategy is shown in Table 1. The search strategy for each database is detailed in Supplementary Table S1.

### 2.4. Study Selection

**Figure 2:**
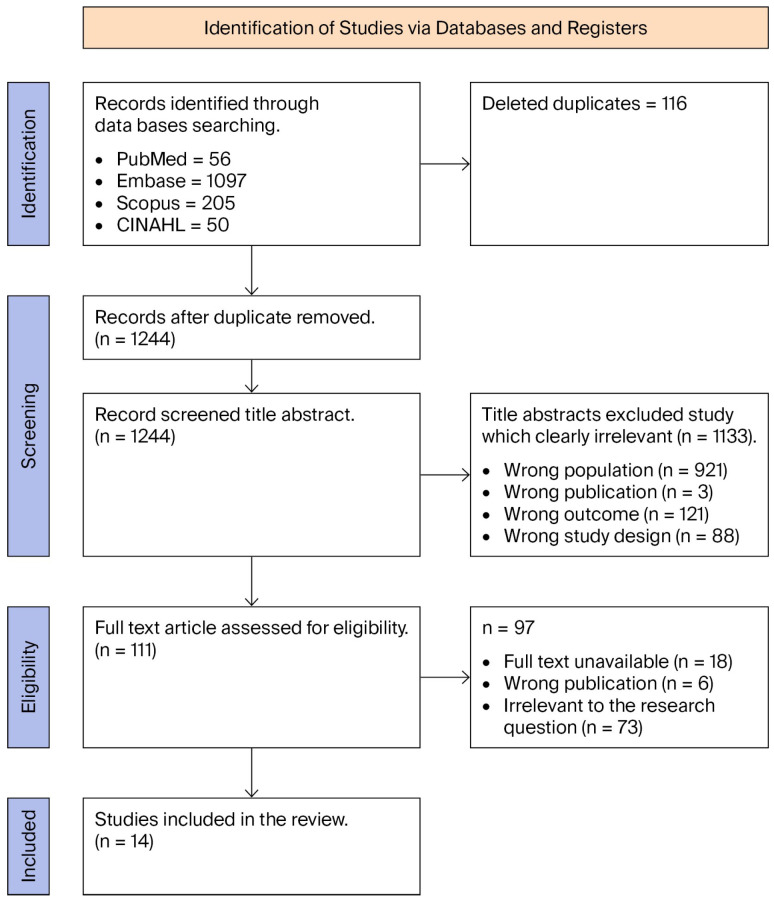
PRISMA flow diagram of included and excluded articles in the role of health literacy of students’ reproductive health.

All studies identified in the screening process were imported into EndNote for duplicate identification and removal. The remaining articles were uploaded to the Rayyan software [35], after which any additional duplicates were removed. The study used a two-level title/abstract screening process. At the first level, three research team members (DAG, LE, and MD) carried out title and abstract screening on the papers that met the inclusion criteria and excluded irrelevant articles through a blinded process. Only articles that were considered ineligible by all authors were eliminated after consultation. All selected articles which met the inclusion criteria were later moved to full text screening using a blinded process. Any disagreements were resolved by a fourth reviewer (VP), who also carried out any final evaluations. Finally, a secondary search, which included reference lists, was conducted to identify any further eligible articles. The process of selection and the stage of each review are shown in Figure 2.

### 2.5. Quality Appraisal

The quality of each study was performed using a mixed methods appraisal tool (MMAT) [36]. This tool was used for its ability to evaluate studies across multiple methodologies, including qualitative, quantitative, mixed method studies, randomized studies and non-randomized studies. The MMAT’s comprehensiveness and precision made it the ideal choice for assessing the diverse methodological studies included in our research. The tool targets many dimensions of bias, which include measurement bias, selection bias, reporting bias, publication bias, and risk of self-reporting bias. Each study was evaluated for bias and result were categorized into one of the three levels: “low risk bias”, “some concern of bias”, and “high level of bias”. This was based on the variables of each study, such as data analysis, sample size, and clear reporting of the methodology. For example, studies with sufficient sample size, well-elaborated methodologies, and comprehensive data analyses and transparency in reporting were categorized as having a low risk of bias. Those that had insufficient sample size and were at possible risk for self-reporting bias but still provided valuable insights were categorized as having some concerns of bias, while those with insufficient sample size, unelaborated reported methodology, inappropriate statistical analyses, or high likelihood of reporting bias were categorized as having a high-level of bias. Table 2 summarizes the distribution of studies across bias categories and demonstrates how many studies were classified as “low risk of bias”, “some concern of bias”, and high level of bias.”

Although MMAT provided a comprehensive framework, some challenges arose during the evaluation process, particularly when interpreting risk of bias in studies with insufficient reporting. To address these challenges, a third reviewer was recruited to resolve any discrepancies through discussion, thus ensuring consistency in the blinded evaluations by the authors. In addition, reviewers used the Strengthening the Reporting of Observational Studies in Epidemiology (STROBE) checklists to determine the relevance, quality, and eligibility of the studies [37]. At the final stage, all the disputes raised were resolved by consensus, and only those studies with no issues were considered to have a low risk of bias and were included in the review.

### 2.6. Data Abstraction and Analysis

The principal researcher developed the data extraction tool used in this systematic review, and a pre-test was performed before the commencement of data extraction by the research teams involved in this systematic review. The data extraction tool was finalized after pretesting and agreement with the research team. Some of the variables included were as follows: title of the study, authors, publication year, study design, country/region, population, sample size, health literacy measure, outcome measure, variable adjustment, results, conclusions, and recommendations.

## 3. Results

### 3.1. Yield of Database Search

The first database search yielded a total of 1360 articles, and after removing 116 duplicate articles through EndNote, a total of 1244 articles were screened for title and abstract screening. A total of 1133 articles were excluded from further screening stages because they addressed the wrong population, had undergone the wrong form of publication (without peer review), or included the wrong type of intervention. Finally, a total of 111 articles proceeded to full text screening, of which 94 articles were again excluded because the full text was missing, because they were protocol papers, because the results were not well reported, or because the wrong study design was used. Finally, a total of 14 articles were included because they met all inclusion criteria and were of good quality, as shown in Figure 2. The characteristics of these studies are elaborated in Table 3.

### 3.2. Characteristics of Included Studies

Table 3 shows that 14 studies were included in this review. The majority (10 studies) used a cross-sectional approach [3,17,21,43,50,51], while other 4 comprised a quasi-experimental study, a mixed methods study, a focus group discussion (FGD), and a descriptive study [52,53]. These studies covered different topics with different themes: Eight studies focused on reproductive and sexual health literacy, fertility awareness, preventive behaviours, childbearing intention literacy, level of sexuality and reproductive health knowledge, HPV knowledge, and information-seeking behaviour [50]. One study examined sexual and reproductive health knowledge, attitudes and behaviours [54], and another looked at the effect of sexual health education on sexual myths and sexual health literacy [3]. Additionally, two studies examined emergency contraception knowledge, contraceptive awareness and use, and e-health literacy [17]. Finally, the two remaining studies looked at HPV misconceptions and HPV knowledge, respectively [55]. Geographically, studies were predominantly conducted in the United States of America (USA) (four); other countries included Turkey (four) and Thailand (two), and the remaining studies were from Poland, Sierra Leon, Indonesia, and Cameroon [38]. Some studies were conducted in university settings, while others were conducted in colleges [17,44]. Some of the studies provided a mean age with standard deviation of (21.95 ± 2.45, 24.3 ± 5.63, 21.98 ± 4.72, 19.6 ± 1.37), while others provided a range or interquartile range (IQR).

### 3.3. Measurement Tools Used in Included Studies

Health literacy measurement varied across the studies; a significant number of the studies used established health literacy assessment tools. One study utilized the Rapid Estimate of Adult Literacy in Medicine (REALM). Additionally, two studies used the Newest Vital Sign (NVS). One study utilized the European Health Literacy Projects Questionnaire (HLS-EU), and one study employed the eHEALS questionnaire. Several studies used self-developed questionnaires or interviews tailored to their specific research questions, such as assessing STI knowledge, emergency contraception knowledge, or knowledge of the female body. Other unique measures included the Sexual and Reproductive Health Knowledge Scale, adaptations of existing tools for adolescents (REALM-Teen and NVS health literacy interview, Kids Poll), the HPV Knowledge Scale (HPV-KS), Turkish Health Literacy, the SHL questionnaire, and the Health Literacy Questionnaire (HLQ). Finally, one study incorporated a Personal Information Form and a Sexual Myths Scale.

### 3.4. The Main Findings from Included Studies

Table 4 shows the findings, which underscore the critical role of health literacy in reproductive health among college and university students. The studies by Ashley Sons and Ann L. Eckhardt and by Ewelina Chawlowska et al. highlight a significant gap in reproductive knowledge, necessitating tailored educational interventions [23,38]. The need for parental education on sexual health literacy was emphasized the research of Eusebius Small et al., and Filiz Aslantekin-Özcoban and Mukadder Gün advocated for counselling and support centres to address the needs of young adults [39,40]. Mereerat Manwong examined the correlation between low scores in sexual health literacy and preventive behaviours, recommending an online educational program to improve knowledge [41]. In addition, Amy E. Albright and Rebecca A. Allen pointed out that misconceptions about HPV can be mitigated through public health education, and Cherly A. Vamos recommended the use of hybrid interventions composed of technology and peer support [27]. Akoku et al. stressed the need for targeted interventions to improve fertility awareness, while Izzatul Arifah calls for integrating health literacy programs into educational curricula [43,44]. Rabia Sohbet and Fatma Gecici proposed establishing youth consultancy units in universities to provide accessible reproductive health education [45].

### 3.5. Health Literacy and Knowledge

Ewelina Chawlowska et al. found that higher health literacy is prevalent among educated women. After assessing students’ knowledge of fertility-related physiology and fertility patterns, they found that older students who were 25 years old and above and medical university students were the most knowledgeable. This research found that while 93.4% of students correctly identified the optimum age for women to have their first child for faster pregnancy, only 47.1% reported gaining information from multiple sources and 28.3% cited primary or middle school classes as their sources [38]. At the same time, Filiz Aslantekin and Mukadder Gun found that emergency contraception knowledge varied significantly by gender. In this study, female students scored an average of 5.7 ± 2.8. In contrast, male students scored 4.3 ± 2.0 (*p* < 0.05), of whom students who received reproductive health education had higher emergency contraceptive knowledge scores (5.8 ± 3.1); however, 59.1% of surveyed students were unfamiliar with the emergency contraceptive [40]. Two of the studies [38,40] emphasized the positive correlation between health literacy and knowledge, for example, fertility and emergency contraceptives.

### 3.6. Influence of Socioeconomic Factors

Eusebius Small et al. and Derick Akompab et al. highlighted the impact of socioeconomic factors on health behaviours. Small, who assessed relationship between sexual health literacy, parental education, and risky sexual behaviour, found that parental health literacy significantly affects students sexual risk behaviours, indicating that family background plays a vital role in shaping health decisions; fathers’ education, in particular, emerged as the strongest contributor to sexual health literacy among college students [39]. Fathers’ education (loading factor = 0.843, *p* = 0.0001) and income (loading factor = 0.695, *p* = 0.0001) were the strongest contributors to sexual health literacy among college students. Similarly, Akoku demonstrated a link between fertility awareness and contraceptive use, suggesting that socioeconomic status and access to information directly influence reproductive health choices. The finding showed that, among the total participants, 99.3% indicated wanting to have children. Only 49.3% knew their fertile period, and 62.5% of the sexually active female university students were current contraceptive users. There was a statistically significant association between fertility awareness knowledge and period abstinence (PR = 1.57; 95% CI: 1.02–2.44, *p* = 0.049) [43]. Another study by Prémusz V. et al. also shows that social-economic inequalities are the main determinant of women’s knowledge about their health [56].

### 3.7. Gender Differences in Health Literacy

The findings of Ashley Sons and Ann L. Eckhardt; Elif Senocak Tasci et al.; and Amy E. Albright and Rebecca S. Allen revealed notable gender differences in health literacy and reproductive health knowledge after examining the health literacy and knowledge of female reproduction, contraception, and STIs [23,42,47]. Sons found that male students exhibited lower knowledge scores than female students. The findings summarized significant differences in health literacy and reproduction knowledge across genders. Almy found that more than half of the participants had received at least one HPV vaccine and health literacy was associated with a greater knowledge of HPV and the availability of vaccines. Familiarity with HPV vaccines was high, but knowledge of the virus itself was lacking. In the other study, Tasci highlighted that women generally have better HPV knowledge. The findings showed that male participants demonstrated significantly lower KFB scores than female participants (*p* < 0.001). In contrast, transgender participants demonstrated lower health literacy and lower knowledge of basic female reproduction than cisgender participants. Health sciences students showed better HPV awareness, with 78% students being aware of HPV compared to 65% of others (*p* = 0.007). All participants demonstrated low knowledge of the menstrual cycle, early physical signs of pregnancy, and certain contraceptives.

### 3.8. Impact of Education Intervention on Health Literacy

The effectiveness of educational programs in enhancing health literacy is evident in the studies of Yağmur Sürmeli et al. and Mereerat Manwong, who evaluated the effect of sexual health education on sexual myths and sexual health literacy in university students, and in other studies, which investigated factors associated with SHL and preventive behaviours [41,49]. Sürmeli demonstrated that sexual health education significantly improved students’ knowledge regarding sexual myths. In this research, they found that before sexual health education, 49% of the students had moderate knowledge about sexuality. After the training, more than half of the students (51%) were found to have sufficient knowledge about sexuality. While Manwong identified sexual health literacy (SHR) as a key factor in promoting preventive behaviours against STDs and unintended pregnancies, his study shows that more than 85% reported having a good or very good relationships with their parents. Approximately 7.3% of the students reported having sexual intercourse, and the average scores of comfortably communicating with their parents about sex issues, the influence of friends and media, and SHL were 20.30 (SD = 5.16), 35.91 (SD = 4.93), and 96.81 (SD = 12.80), respectively [41].

### 3.9. Technology and E-Health Literacy

The role of technology in enhancing health literacy was emphasized by Filiz Aslantekin-Özcoban and Mukadder Gün and by Cheryl A. Vamos in systematic reviews. Aslantekin-Özcoban’s findings showed that e-health literacy is positively correlated with knowledge of emergency contraception, suggesting that digital platforms can be powerful tools for disseminating reproductive health information [27,40]. Vamos’s research supports this by identifying the internet as a primary source for sexual and reproductive health information. Participants in these studies discussed facilitators and barriers to understanding SRH information, indicating the need for improved health literacy skills. The authors came to this conclusion after they investigated university students’ knowledge of emergency contraception, influencing factors, and e-health literacy levels and explored college students’ sexual and reproductive health literacy, respectively.

### 3.10. Cultural and Contextual Influences

The studies by Rabia Sohbet and Fatma Gecici and by Izzatul Arifah highlight the importance of cultural context in shaping health literacy and reproductive health outcomes [44,45]. Sohbet found that older students had better sexual and reproductive health knowledge, indicating that cultural norms and educational experiences influence health literacy levels. Among the study participants, 83.3% wanted SRH included in the school curriculum, 54.5% discussed SRH issues with friends, and 80.2% desired a consultancy unit at the university. The findings showed a strong desire for comprehensive SRH education among the students. Arifah’s research underscores that health literacy significantly impacts the utilization of reproductive health services among adolescents, suggesting that the cultural acceptance and understanding of these services are critical for effective health outcomes. These findings showed that the probability of utilizing reproductive health counselling services doubled for students with good literacy levels (OR = 2.1, 95% CI 1.01–4.49).

### 3.11. Health Literacy Levels and Associated Behaviours

Nearly half of the participants had low health literacy levels, which were associated with unhealthy behaviours and lower self-rated health, as reported by A. Esoon Park et al. Other researchers, Premyuda Narkarat, MNS, and Surasak Taneepanichskul, MD, found that GPA was significantly associated with sexual health literacy, suggesting that academic performance may reflect or influence health literacy level.

## 4. Discussion

This systematic review showed that higher health literacy is associated with an enhanced understanding of important reproductive health topics [14,18,23,27,43,44,57]. Health literacy plays an important role in improving the reproductive health knowledge and behaviours of college and university students [27]. This suggests that while some students possess foundational knowledge, critical areas remain underexplored, particularly factors affecting reproductive health.

Several studies identified significant gaps in college and university students’ knowledge regarding reproductive health. A study written by Ewelina Chawlowska et al. found that while older students and medical university students demonstrated a strong understanding of fertility-related issues, there was poor awareness of the adverse effects of unbalanced diets and psychological distress. Health literacy can be improved by emphasizing the need for relevant courses to enhance reproductive health knowledge among professionals, stressing the importance of considering health literacy levels when educating college students, advocating for educational intervention, and suggesting that age-appropriate sexual health education can improve knowledge and dispel myths related to reproductive health, thereby enhancing health literacy [23,38,47,49]. The desire for more comprehensive reproductive health is clear, as noted by Rabia Sohbet and Fatma Gecici, where a significant percentage of students expressed the need for reproductive health education to be included in school curricula. This aligns with the findings of Yağmur Sürmeli et al., where students demonstrated increased knowledge following health education. Implementing structured reproductive health programs in schools could address knowledge gaps and foster healthier attitudes toward sexual health.

The relationship between health literacy and reproductive health behaviours is critical. Derick Akompab Akoku et al. found a significant association between fertility awareness and contraceptive use, indicating that improved health literacy can lead to better reproductive health outcomes. Izzatul Arifah’s research indicated that students with higher health literacy are more likely to utilize reproductive health services, and Filiz Aslantekin-Özcoban and Mukadder Gün highlighted that a substantial percentage of students lacked knowledge about emergency contraceptives, with 59.1% being unfamiliar with the concept. This points to the potential for comprehensive health literacy programs to empower students in making informed decisions about their reproductive health. A Esoon Park et al. reported that almost half of the students had low health literacy levels, which were associated with unhealthy behaviours and lower self-rated health, while Premyuda Narkarat, MNS, and Surasak Taneepanichskul, MD, found that students’ GPA was significantly associated with reproductive health literacy, suggesting that academic performance may reflect or influence health literacy levels. Another study performed by Elif Şenocak Taşçı et al. on the assessment of health literacy and HPV knowledge found that health science students had a significantly higher awareness of HPV compared to their peers. This indicates that specific educational backgrounds can enhance knowledge of a particular health issue.

Gender disparities in health literacy are evident across multiple studies, for instance, research by Amy E. Albright and Rebecca S. Allen and by Ashley Sons and Ann L. Eckhardt found that female participants had higher health literacy regarding HPV than their male counterparts and that male participants scored lower on knowledge of reproductive health. These differences reflect broader societal norms and expectations regarding gender roles in health education. Tailoring educational materials to address these disparities could enhance engagement and understanding among all genders. Derick Akompab Akoku et al. emphasized the need for targeted interventions to improve fertility awareness and reproductive health knowledge among female students, and another author, A. Esoon Park et al., also pointed out the need for improved health literacy in high school students to reduce risky behaviours [43,46]. The gender disparities in health literacy underscore the necessity of targeted educational interventions to improve male students’ understanding of reproductive health issues [23,47].

The study performed by Eusebius Small et al. underscores the role of socioeconomic factors in shaping health literacy by establishing the association between parental education and reproductive health knowledge. The study found that higher paternal education levels were significantly linked to better reproductive health literacy among college students. This suggests that interventions to improve health literacy should consider familial and socioeconomic contexts, potentially incorporating family education as a component of a reproductive health program. Socioeconomic factors significantly influence the health literacy of students [56], and supportive educational backgrounds foster healthier choices among students [39,43]; additionally, understanding cultural context is important when influencing health literacy and service utilization [44,45]. A study performed by Prémusz V et al. also shows social concern and relationship concern to be some of the factors that affect health literacy and infertility [56,58]. A recent scoping review by Alhussaini et al. confirmed that sexual and reproductive health literacy is influenced by complex factors, including age, gender, religious and cultural background, and family and school socialization. Effective interventions should therefore not only be individual-based but also take into account the social and environmental context [57].

The use of informal sources of information, such as friends and the internet, as highlighted by Cheryl A. Vamos, raises a concern about the accuracy and reliability of the information students receive. This study showed that, while the internet is a predominant source of reproductive health information, barriers, such as medical jargon, hinder understanding. This emphasizes the need for educational interventions that not only provide accurate information but also teach critical appraisal skills for evaluating online resources. Some of the educational intervention methods recommended by this systematic review are equipping college and university students with accurate sex education, considering parental influence, creating call centres for counselling and support centres to meet young people’s sexual and reproductive health needs, and advocating for hybrid interventions involving technology and peer support to enhance reproductive health decision-making [27,39,40]. Additionally, the integration of technology into health education serves as a powerful tool for the dissemination of reproductive health information [27]. The effectiveness of educational programs in increasing health literacy can lead to healthier sexual behaviour [41,49].

Addressing misconceptions and gaps can enhance health literacy regarding reproductive health among students. This can be achieved by identifying the misconceptions and the need for public health education to increase knowledge [42], with significant gaps noted in sexual health literacy and preventive behaviour; by adopting online programs for learning [41]; and by comprehensive skills development in accessing and applying health information [48]. Improving peer education and supporting the educational system can be achieved by establishing peer training programs and youth consultancy units in universities to enhance sexual and reproductive health education [45]. One of the studies, by Izzatul Arifah et al., stresses the importance of integrating health literacy programs into school curricula to motivate adolescents to utilize reproductive health services [44].

## 5. Conclusions

This systematic review study highlights significant gaps in sexual and reproductive health literacy among young people, particularly female students and adolescents, with many exhibiting low levels of knowledge and prevalent misconceptions regarding contraception, fertility, HPV, and preventive behaviours. Several authors emphasize the need for comprehensive, age-appropriate, and culturally sensitive educational interventions, including graduate and postgraduate in-services training, online programs, and hybrid methods, to equip both professionals and students with accurate knowledge. Parental education and involvement are identified as influential factors shaping sexual health literacy behaviours. The establishment of accessible counselling and support services within educational institutions is frequently recommended to promote healthy sexuality and improve e-Health literacy. Improved health literacy is linked with the better utilization of reproductive health services, increased HPV vaccine uptake, and safer sexual behaviours. Gender and demographic factors also affect literacy levels, suggesting that tailored interventions targeting diverse populations may be more effective. Overall, the studies call for targeted, multi-level approaches integrating technology, formal education, counselling, and community involvement to enhance sexual and reproductive health literacy and empower informed decision-making among young people (see Table S2).

## 6. Limitations

A limitation of this study was the small sample sizes in some of the included studies, which may impact the generalizability of the findings. Additionally, the research was limited to articles written in English, potentially excluding valuable insights from non-English sources. The focus on specific population groups with distinct characteristics may restrict the applicability of the results to more diverse populations, thus creating potential publication bias. Addressing the above limitations in future research is essential for enhancing the robustness and applicability of the results.

## Figures and Tables

**Table 1:** Search strategy.

Search #	Search Terms
#1	College Student* OR Student* OR University* OR Universities OR Higher Education OR Campus
#2	Health Literacy
#3	Fertility OR Fertility Awareness OR Fertility Knowledge OR Fertility Intentions OR Fecundability OR Fecundity OR Fertility Incentives OR Fertility Incentive OR World Fertility Survey OR Fertility Determinants OR Fertility Preferences OR Fertility Preference OR Reproductive Behaviour OR Childbearing OR Childbirth OR Reproductive Health Knowledge OR Fertility Education OR Conception Knowledge OR Reproductive Life Span OR Pregnancy Planning OR Fertility Literacy OR Reproductive Health
#4	#1 AND #2 AND #3

**Table 2:** Bias categories across all studies.

MMAT Parameters	Low Risk of Bias	Some Concern of Bias	High Level of Bias
Strong methodology with a substantial sample size. A clear description of variables and control for confounders. Robust statistical analysis.	10	0	0
Well-described methodology with sufficient sample size and comprehensive statistical analysis.	2	0	0
Clear methodology, large sample size, valid measurement tools, and proper statistical analysis conducted.	0	0	0
Small sample size, clearly described methods, and possible risk of self-reporting bias present.	0	2	0
Small sample size and unelaborated methodology.	0	0	0
Sufficient data, inappropriate data analysis, and unclear reporting methodology.	0	0	0
Total Studies	12	2	0

**Table 3:** Characteristics of studies included in the review.

First Author	Study Design	Aim	Country	Topic	Age
Ewelina Chawłowska et al., 2020 [38]	Cross-sectional study	Assessing student’s knowledge related to fertility physiology and fertility patterns	Poland	Reproductive health literacy and fertility awareness	21.95 ± 2.45
Eusebius Small et al., 2023 [39]	Cross-sectional study	Assessing the relationship between sexual health literacy, parental education, and risky sexual behaviour	Sierra Leon	Sexual health literacy, parental education, and risky sexual behaviour	24.3 ± 5.63
Aslantekin-Özcoban F, Gün M 2021 [40]	Cross-sectional study	Assessing university student knowledge on emergency contraception, influencing factors and e-health literacy levels	Turkey	Emergency contraception knowledge level and e-health literacy	
Mereerat Manwong 2022 [41]	Mixed methods study	Investigating factors associated with SHL and preventive behaviours	Thailand	Sexual health literacy and preventive behaviours	
Amy E. Albright, Rebecca S. Allen 2018 [42]	Cross-sectional study	Assessing HPV knowledge and awareness	USA	HPV misconceptions and the role of health literacy	
Ashley Sons and Ann L. Eckhardt, 2023 [23]	Cross-sectional study	Examining health literacy and knowledge of female reproduction, contraception, and sexually transmitted infections (STIs)	USA	Health literacy and knowledge of female reproduction in undergraduate students	19.9 ± 1.2
Derick Akompab Akoku et al., 2022 [43]	Cross-sectional study	Examining the association between fertility awareness knowledge, and contraceptive use	Cameroon	Childbearing intentions, fertility awareness knowledge, and contraceptive use	23 years (IQR = 21–25)
Cheryl A. Vamos, 2020 [27]	Focus Group Discussion	Assessing college students’ sexual and reproductive health (SRH) literacy experiences, specifically contraception use and STI prevention	USA	Exploring college students’ sexual and reproductive health literacy	
Izzatul Arifah 2022 [44]	Cross-sectional study	Examining the relationship between health literacy and use of reproductive health counselling services among high school students	Indonesia	Health literacy and utilization of reproductive health services	
Rabia Sohbet Fatma Gec ici, 2014 [45]	Descriptive study	Examining the level of knowledge on sexuality and reproductive health (SRH)	Turkey	Examining the level of knowledge on sexuality and reproductive health	
Park A et al., 2017 [46]	Cross-sectional study	Assessing health literacy using three validated measures and examining cross-sectional and prospective associations between health literacy and adolescent health behaviours and outcomes	USA	Associations between health literacy and health behaviours	14 years
Elif Şenocak Taşçı, MD et al., 2023 [47]	Cross-sectional study	Assessing university students’ knowledge of HPV and the association between HL and HPV vaccinations	Turkey	Assessment of health literacy and HPV knowledge	21.98 ± 4.72
Narkarat and Taneepanichskul MD 2021 [48]	Cross-sectional study	Assessing the level of SHL and exploring factors associated with SHL	Thailand	Factors associated with sexual health literacy	
Yağmur Sürmeli et al., 2024 [49]	Quasi-experimental	Evaluating the effect of sexual health education sexual myths and sexual health literacy in university students	Turkey	The effect of sexual health education on sexual myths and sexual health literacy	19.8 ± 1.37

**Table 4:** Results of individual studies included in the analysis.

Author	Sample Size	Outcome (Measure Type)	Variables of Adjusted Analysis	Result
Ewelina Chawłowska et al., 2020 [38]	456 women	Assess student’s knowledge fertility-related physiology and fertility patterns	Knowledge, age, year of study, university, sources of information	Health literacy is associated with different knowledge levels; higher levels were observed in women, people with higher education, those having difficult conceiving, and those who had planned their pregnancies
Eusebius Small et al., 2023 [39]	338 university students	Relationship between sexual health literacy, parental education, and risky sexual behaviour	Family social economic status (SES), STI knowledge, sexual risk behaviour	Parental health literacy is significantly associated with student’s sexual risk behaviour
Aslantekin-Özcoban F, Gün M 2021 [40]	1003 senior undergraduate students	Assess university student knowledge of emergency contraception, influencing factors, and e-health literacy levels		Knowledge of emergency contraception were significantly correlated positively with e-Health literacy; therefore, improving e-health literacy of students can be key to improving knowledge of emergency contraception
Mereerat Manwong 2022 [41]	730 students; 59.2% female and 40.8% male	Investigate factors associated with SHL and preventive behaviours among middle school students	Sex, nightlife, drinking alcohol beverages, sexual intercourse experience and sexual health literacy	Significant factors associated with preventive behaviours regarding pregnancy and STDs were sex, nightlife venue, drinking alcoholic beverages, sexual intercourse experience, and SHL. The most effective factor was SHL, which was the main concept integrated into the online program
Amy E. Albright, Rebecca S. Allen 2018 [42]	360 students from South-Eastern University in the USA	Assess HPV knowledge and awareness in a sample of US college students		Health literacy was not related to vaccination status; it was associated with a greater knowledge of both HPV and available vaccines. The sociocultural aspect of health literacy was also found to significantly relate to HPV knowledge
Ewelina Chawłowska et al., 2020 [38]	456 women	Assess student’s knowledge of fertility-related physiology and fertility patterns	Knowledge, age, year of study, university, sources of information	Health literacy is associated with different knowledge levels, higher level in women, people of higher education, those having difficult conceiving, and those who had planned their pregnancies
Park A. et al., 2017 [46]	250 adolescents	Assess health literacy using three validated measures and examine cross-sectional and prospective associations between health literacy and adolescent health behaviours and outcomes	Age, male, White ethnicity, free lunch program	Health literacy was associated with a lower self-rating of general health, unhealthier diet, heavier weight, and greater engagement in problem behaviours and sexual behaviours at baseline. Lower baseline health literacy also was associated with a greater increase in substance use over time
Elif Şenocak Taşçı, MD et al., 2023 [47]	361 university students	Assess the university students’ knowledge about HPV and the association between HL and HPV vaccination		General HPV knowledge level was significantly better among women and those with a family history of cancer and was significantly lower among students in their prep or first year of school. Higher levels of HPV knowledge and total HPV-KS score were statistically significantly higher in students with adequate/excellent HL
Narkarat and Taneepanichskul MD 2021 [48]	128 females secondary school students	Assess the level of SHL and to explore factors associated with SHL		The results showed that the grade point average (GPA) was statistically significantly associated with SHL
Yağmur Sürmeli et al., 2024 [49]	51 students; 84.3% female, 15.7% male	Evaluate the effect of sexual health education on university students’ understanding of sexual myths and sexual health literacy		Increased knowledge after training

## Data Availability

No new data were created or analyzed in this study.

## Supplementary Materials

The following supporting information can be downloaded at: https://www.mdpi.com/article/10.3390/healthcare13182342/s1, Table S1: Full search strategy for each database; Table S2: summary of conclusions and recommendations.

## Abbreviations

The following abbreviations are used in this manuscript:

PROSPERO	Prospective Register for Systematic Reviews
REALM	Rapid Estimate of Adult Literacy in Medicine
HLQ	Health Literacy Questionnaire
PRISMA	Reporting Items for Systematic Reviews and Meta-Analyses
IQR	Interquartile range
HLS-EU	European Health Literacy Project Questionnaire
NVS	Newest Vital Sign
FA	Fertility Awareness
STROBE	Strengthening the Reporting of Observational Studies in Epidemiology
MMAT	Mixed Methods Appraisal Tool
