# Supplementary material for: Exploring the Impact of Health Literacy on Fertility Awareness and Reproductive Health in University Students—A Systematic Review
Source: Healthcare (Basel). 2025 Sep 17;13(18):2342. doi: 10.3390/healthcare13182342 (PMC12469965; doi:10.3390/healthcare13182342)
Supplement: Supplementary file 1 [file healthcare-13-02342-s001.zip › healthcare-3797728-supplementary.pdf]

Table S1: Full search strategy for each database

Data base	Search strategy	Search result
PubMed	((((((((((((((((((((((Fertility[MeSH Terms]) OR (Fertility[Title/Abstract])) OR ("Fertility Awareness"[Title/Abstract])) OR ("Fertility Knowledge"[Title/Abstract])) OR ("Fertility Intentions"[Title/Abstract])) OR (Fecundability[Title/Abstract])) OR (Fecundity[Title/Abstract])) OR ("Fertility Incentives"[Title/Abstract])) OR ("Fertility Incentive"[Title/Abstract])) OR ("World Fertility Survey"[Title/Abstract])) OR ("Fertility Determinants"[Title/Abstract])) OR ("Fertility Preferences"[Title/Abstract])) OR ("Fertility Preference"[Title/Abstract]))) OR ("Reproductive Behavior"[MeSH Terms])) OR ("Childbearing"[Title/Abstract])) OR (Childbirth[Title/Abstract])) OR ("Reproductive Health Knowledge"[Title/Abstract])) OR ("Fertility Education"[Title/Abstract])) OR ("Conception Knowledge"[Title/Abstract])) OR ("Reproductive Life Span"[Title/Abstract])) OR ("Pregnancy Planning"[Title/Abstract])) OR ("Fertility Literacy"[Title/Abstract])) OR (Reproductive health[Title/Abstract])	56
Scopus	(TITLE-ABS-KEY ("college student*" OR student* OR university OR universities OR "Higher Education" OR campus)) AND (TITLE-ABS-KEY ("Health Literacy")) AND (TITLE-ABS-KEY (fertility OR "Fertility Awareness" OR "Fertility Knowledge" OR "Fertility Intentions" OR fecundability OR fecundity OR "Fertility Incentives" OR "Fertility Incentive" OR "World Fertility Survey" OR "Fertility Determinants" OR "Fertility Preferences" OR "Fertility Preference" OR "Reproductive Behavior" OR childbearing OR childbirth OR "Reproductive Health Knowledge" OR "Fertility Education" OR "Conception Knowledge" OR "Reproductive Life Span" OR "Pregnancy Planning" OR "Fertility Literacy" OR "Reproductive Health"))	105
CINAHL	College Students Or Students Or Undergraduates Or Higher Education Or Campus AND Health Literacy Or Health Education Or Health Knowledge Or Health Information Or Health Understanding AND Fertility OR Fertility OR Fertility Awareness OR Fertility Knowledge OR Fertility Intentions OR Fecundability OR Fecundity OR Fertility Incentives OR Fertility Incentive OR World Fertility Survey OR Fertility Determinants OR Fertility Preferences OR Fertility Preference OR Reproductive Behavior OR Childbearing OR Childbirth OR Reproductive Health Knowledge OR Fertility Education OR Conception Knowledge OR Reproductive Life Span OR Pregnancy Planning OR Fertility Literacy OR Reproductive Health	50
Google Scholar	Health Literacy, Fertility Awareness, Reproductive Health, University Students	37
Embase	'college student'/exp OR 'college student' OR 'student'/exp OR 'student' OR 'university student'/exp OR 'university student' OR 'university'/exp OR 'university' OR 'tertiary education'/exp OR 'tertiary education' OR 'campus'/exp OR 'campus' AND 'health literacy' AND 'birth control' OR 'infertility therapy' OR 'fertility incentives' OR 'fecundability' OR 'fertility' OR 'world fertility surveys' OR 'fertility determinants' OR 'fertility preferences' OR 'reproductive behavior' OR 'reproductive behavior determinants' OR 'pregnancy' OR 'childbirth education' OR 'childbirth' OR 'reproductive health survey' OR 'reproductive health' OR 'contraception' OR 'conception assistance device' OR 'reproductive lifespan' OR 'pregnancy planning' OR 'health service'	1097
Others		18
Total		1360

Table S2. The Conclusion and Recommendation from Selected Studies.

No	Author	Conclusions and recommendations
1	Ewelina Chawłowska et al. 2020 (49)	Reproductive health knowledge among the young female students is incomplete, relevant graduate, postgraduate and in-service courses should be available to professionals responsible for spreading reproductive health knowledge
2	Eusebius Small et al. 2023 (51)	Parental education in shaping sexual health literacy and ultimately influencing risky sexual behaviours among college students. Equip college students with accurate sex education, consideration role of parental education in this context
3	Aslantekin-Özcoban F, Gün M 2021 (50)	There is a need for services that support healthy sexuality in adolescence and young adults, including the establishment of counselling and support centres. Improved e-health literacy among students can enhance their knowledge
4	Mereerat Manwong 2022 (52)	The finding of study shows low scores in sexual health literacy (SHL) and preventive behaviours for pregnancy and STDs, with key influencing like gender. Recommends that engaging online programs and diverse methods and tools
5	Amy E. Albright, Rebecca S. Allen 2018 (55)	College students hold serious misconceptions about HPV that may be redressed through public health education programs to increase health literacy and knowledge. Public health interventions would potentially increase HPV vaccine uptake, leading to decreased cervical cancer incidence and mortality rates.
6	Ashley Sons & Ann L. Eckhardt, 2017	Undergraduate students have major reproductive knowledge gaps. Healthcare providers need to consider health literacy and knowledge level when educating college students.
7	Derick Akompab Akoku et al. 2022 (40)	Most of female students intend to have children in the future, but their fertility awareness knowledge was lacking. There was a statistically significant relationship between fertility awareness knowledge, and the use of male condoms and the withdrawal method, emphasizing the need for targeted interventions to improve their understanding
8	Cheryl A. Vamos, 2015	The result shows that hybrid intervention combining technology and informed individuals can enhance students' sexual and reproductive health decision making. Therefore, improving health literacy can reduce unintended pregnancies
9	Izzatul Arifah 2022 (48)	The outcome shows adolescent health literacy level is associated with the utilization of reproductive health services. There is need for further research and school-based intervention is needed to improve adolescent's health literacy
10	Rabia Sohbet • Fatma Gec ici, 2014 (53)	Students exhibited medium knowledge of sexual and reproductive health, with higher scores among females, fourth year students, the mostly relay friends for information. University should establish accessible consultancy units
11	Park A et al. 2017 (57)	Lower health literacy was associated with lower general health, heavier weight status, and greater engagement in problem behaviors. Results point to the pressing need to improve health literacy in urban high school students
12	Elif Şenocak Taşçı, MD et al. 2023 (58)	Result shows that knowledge of HPV vaccination is closely related to HL level, therefore, education interventions on HL may be considered to increase HPV vaccination rates.
13	Narkarat and Taneepanichskul MD 2021 (59)	Majority of student in the study have inadequate scores on SHL. Therefore, knowledge information on SHL should be provided and encouraged among high school teenagers
14	Yağmur Sürmeli, et al. 2024 (60)	Students sexual health knowledge increased from 49% to 51% after training, with reduction in myth score. Age-appropriate education should be provided by trained professionals in educational institutions
